# Uptake of Plasmin-PN-1 Complexes in Early Human Atheroma

**DOI:** 10.3389/fphys.2016.00273

**Published:** 2016-06-30

**Authors:** Kamel Boukais, Richard Bayles, Luciano de Figueiredo Borges, Liliane Louedec, Yacine Boulaftali, Benoit Ho-Tin-Noé, Véronique Arocas, Marie-Christine Bouton, Jean-Baptiste Michel

**Affiliations:** ^1^UMR 1148, Laboratory for Vascular Translational Science, Institut National de la Santé et de la Recherche MédicaleParis, France; ^2^Paris7 Denis Diderot UniversityParis, France; ^3^Department of Physiology and Pharmacology, Oregon Health and Science UniversityPortland, OR, USA; ^4^Departement of Biological Science, Federal University of São PauloSão Paulo, Brazil; ^5^Heart Institute (InCor), Hospital das Clínicas da Faculdade de Medicina da Universidade de São PauloSão Paulo, Brazil

**Keywords:** vascular smooth muscle cells, proteases, antiproteases, endocytosis, and atherosclerosis

## Abstract

Zymogens are delivered to the arterial wall by radial transmural convection. Plasminogen can be activated within the arterial wall to produce plasmin, which is involved in evolution of the atherosclerotic plaque. Vascular smooth muscle cells (vSMCs) protect the vessels from proteolytic injury due to atherosclerosis development by highly expressing endocytic LDL receptor-related protein-1 (LRP-1), and by producing anti-proteases, such as Protease Nexin-1 (PN-1). PN-1 is able to form covalent complexes with plasmin. We hypothesized that plasmin-PN-1 complexes could be internalized via LRP-1 by vSMCs during the early stages of human atheroma. LRP-1 is also responsible for the capture of aggregated LDL in human atheroma. Plasmin activity and immunohistochemical analyses of early human atheroma showed that the plasminergic system is activated within the arterial wall, where intimal foam cells, including vSMCs and platelets, are the major sites of PN-1 accumulation. Both PN-1 and LRP-1 are overexpressed in early atheroma at both messenger and protein levels. Cell biology studies demonstrated an increased expression of PN-1 and tissue plasminogen activator by vSMCs in response to LDL. Plasmin-PN-1 complexes are internalized via LRP-1 in vSMCs, whereas plasmin alone is not. Tissue PN-1 interacts with plasmin in early human atheroma via two complementary mechanisms: plasmin inhibition and tissue uptake of plasmin-PN-1 complexes via LRP-1 in vSMCs. Despite this potential protective effect, plasminogen activation by vSMCs remains abnormally elevated in the intima in early stages of human atheroma.

## Introduction

The arterial wall is the target of plasma-borne components such as low density lipoprotein (LDL) (Tabas et al., 2007) and circulating zymogens (Lacolley et al., 2012). Like LDL, circulating zymogens and proforms are delivered to the adjacent arterial wall by radial transmural hydraulic conductance (Caro and Lever, 1984; Caro, 2009). In addition to their role in clot lysis, tumoral cell mobilization, and metastasis (Didiasova et al., 2014), enzymes of the fibrinolytic system are involved in the chronic evolution of the plaque at the initial stage of atherosclerotic disease (Torzewski et al., 2004), including migration and loss of vascular smooth muscle cells (vSMCs) by pericellular proteolysis (Meilhac et al., 2003). Plasminogen can be activated on the vSMC membrane platform by t-PA (tissue plasminogen activator) and u-PA (urokinase) release (Borges et al., 2010). vSMCs also produce antiproteases to protect the vascular wall from proteolytic injury (Gomez et al., 2013).

Among these antiproteases, significant quantities of serpins, such as Protease Nexin-1 (PN-1) (Bouton et al., 2003) and plasminogen activator inhibitor-1 (PAI-1), in parallel with tissue inhibitors of metalloproteinases (TIMPs), are known to be expressed by vSMCs (Bouton et al., 2012). PN-1 has emerged as an important actor in the regulation of proteolytic tissue degradation since it is a powerful inhibitor of several serine proteases, including thrombin, plasminogen activators (u-PA, t-PA), and plasmin. PN-1 is able to limit the pericellular activity of plasmin by protecting vSMCs against cell detachment and death (Meilhac et al., 2003; Rossignol et al., 2004). PN-1, a tissue serpin, is poorly diffusible due to its retention by glycosaminoglycans (GAGs) at the cell surface, thereby being barely detectable in the plasma (Bouton et al., 2012). PN-1 is synthesized and secreted under basal conditions by vSMCs, where its expression is up-regulated by mechanical load (Bouton et al., 2003) and transforming growth factor β (TGFβ) (Gomez et al., 2013) or down-regulated by thrombin (Richard et al., 2004). Overexpression of PN-1 limits spreading and migration of vSMCs (Richard et al., 2006). Platelets and phagocytes are also important sources of PN-1 in intraplaque hemorrhages (Mansilla et al., 2008), able to limit fibrinolysis within a thrombus (Boulaftali et al., 2011). Moreover, PN-1 forms covalent complexes with its target proteases, which are cleared by endocytosis, mainly via the LDL receptor-related protein-1 (LRP-1), a member of the scavenger receptor family (Knauer et al., 1997, 1999; Muhl et al., 2007; Lillis et al., 2008).

LRP-1 is a multifunctional cell surface receptor expressed in a wide range of cells, including vSMCs (Moestrup et al., 1992) and macrophages (Lillis et al., 2015). LRP-1 is implicated in endocytosis and the uptake of multiple ligands via its extracellular domain, and signal transduction via its intracytoplasmic domain (Boucher and Gotthardt, 2004). Its large variety of extracellular ligands and cytoplasmic signaling means that LRP-1 plays a role in arterial wall physiology and pathology (Emonard et al., 2014). Specific knockout of LRP in vSMCs (LRP^smc−∕−^) on an LDL receptor (LDLR)^−∕−^ background in mice is highly pathogenic, leading to an exacerbation of atherosclerosis (Boucher et al., 2003, 2007). LRP-1 is also a scavenger receptor responsible for the uptake of LDL, especially aggregated LDL, leading to intracellular accumulation of lipids and transformation of vSMCs (Llorente-Cortes et al., 2006, 2007) and monocyte-derived macrophages (Llorente-Cortes et al., 2007; Lillis et al., 2015) into foam cells in human atheroma.

Our hypothesis is that protease-PN-1 complexes could be engulfed via LRP-1 in foam cells. This mechanism of protease-antiprotease uptake and its consequences have not yet been extensively explored in the human arterial wall *ex vivo* and particularly at the early stage of atheroma. The aim of the present study was to investigate the internalization of plasmin-PN-1 complexes by vSMCs in early human atheroma via the LRP-1 scavenger receptor. A unique collection of early stages of human atheroma, involving normal aorta, fatty streaks, and fibrolipidic initial plaques, provides us the opportunity to decipher plasmin activation and the internalization of plasmin-PN-1complexes in this context.

## Materials and methods

### Patients and aortic specimens

The atherosclerotic samples (*n* = 31) and healthy aortas (*n* = 12) were obtained from anonymous deceased organ donors with the authorization of the French Biomedicine Agency (PFS 09-007) and in accordance with the Declaration of Helsinki. The clinical data associated with this series of patients are reported in Table 1. The healthy aortas were macroscopically devoid of early atheroma, whereas the others presented fatty streaks (FS) or fibrolipidic lesions (FL), defined by subendothelial accumulation of lipids (yellow), capped (FL) or not (FS) by areas of cell proliferation (which appear white). Aortic tissue preparation consisted of an immediate macroscopic dissection to remove the adventitial layer and to separate intima from media, followed by either direct freezing or enzymatic digestion for vSMC primary cultures.

**Table 1:** **Clinical characteristics of patients**.

	**Healthy aorta**	**Early atheroma**	
		**FS**	**FL**
*N*	12	18	13
Age (years)	48.7 ± 14.3	57.9 ± 19.2	65.6 ± 13.9
Sex, % male	70	39	54

*The cohort of patients studied presented 2 groups: patients with a healthy aorta and those with early atheroma. FS: fatty streaks, FL: fibrolipidic lesions. Results are expressed as means ± SD*.

### Aortic tissue extracts and conditioned medium

Extractions (mRNA, protein) were performed from frozen aortic media and intimal tissue after microdissection. The aortic layers were first pulverized in liquid nitrogen using a Freezer mill. Between 20 and 200 mg of aortic powder was used for qPCR and immunoblotting. The numbers of medial and intimal tissue extracts were: healthy aortas (control): *n* = 7, intimal FS: *n* = 12, medial FS: *n* = 9, intimal FL: *n* = 9, medial FL: *n* = 9.

Conditioned medium was obtained by incubation of small pieces of healthy media, intima or media of FS and FL (24 h at 37°C) in a standardized volume (6 ml/g of sample wet weight) of RPMI culture medium supplemented with antibiotics and antimycotics.

### Human vSMC cultures/LDL preparation

Samples of healthy aortic media were incubated for 3 h at 37°C in 0.3% collagenase (LifeTechnologies, Gif-sur-Yvette, France) and 0.1% elastase (Worthington Biochemical Corporation, Lakewood, New Jersey, USA) mixture to obtain primary cultures of aortic vSMCs.

Cells were cultured in SMC Medium 2 from PromoCell (Heidelberg, Germany) with 5% fetal calf serum (FCS). Passages 3 to 4 were used for experiments.

First, vSMCs were starved for 5 h and then incubated for 48 h with LDL or ag (aggregated) LDL at 100 μg/mL in serum-free medium. Cells were then washed with PBS and plasminogen was added to perform plasminogen activation and plasmin assays for 48 h, or proteins were extracted for immunoblotting. Human LDL particles were obtained from pooled plasma of normocholesterolemic volunteers and isolated by sequential ultracentrifugation. The native LDL was conserved in the presence of antioxidants to avoid oxidation before addition to the cells. agLDL was generated by vortexing LDL as previously described (Llorente-Cortes et al., 2000).

### Immunohistochemistry

Atherosclerotic and healthy aortic tissue sections were obtained from paraformaldehyde-fixed, non-complicated plaques, and healthy wall as previously described (Mansilla et al., 2008). Sections were treated for antigen retrieval by heating in a microwave in citrate buffer, pH 6 and the endogenous peroxidase quenched with 3% hydrogen peroxide. Non-specific binding was blocked using PBS-Tween 20 (PBS-T) with 5% BSA for 1 h at room temperature.

Normal and atherosclerotic aortas were incubated overnight with the following antibodies: mouse anti-human PN-1 (1:800, in house Mansilla et al., 2008), goat anti-human LRP-1 (1:80, Santa Cruz Biotechnology, Dallas, Texas, USA), mouse anti-human plasminogen (1:80, Santa Cruz Biotechnology), rabbit anti-human t-PA (1:30, Santa Cruz Biotechnology), rabbit anti-human annexin A2 (1:50, Santa Cruz Biotechnology), goat anti-human P-Selectin (1:50, Santa Cruz Biotechnology), mouse anti-human CD68 (1:100, Dako, les Ulis, France), goat anti-human SM22α (1:200, Abcam, Paris, France), and mouse anti-human α actin (1:50, Dako).

All slides were incubated with secondary antibody conjugated with peroxidase (LSAB-DAKO Kit) for 30 min and revealed by H2O2-dependent oxidation of DAB (3, 3′-diaminobenzidine) precipitation (brown).

The slides were counterstained with Mayer's hematoxylin. For the negative controls, the primary antibody was replaced by an isotype-matched non-specific IgG at the same concentration. As plaques are autofluorescent at multiple wavelengths, it is difficult to discriminate specific signals from the autofluorescent background. Therefore, double fluorescent immunohistochemical staining was not performed in the present study.

### Real-time qPCR

Total RNA was extracted from frozen samples of aortic intima and media (Healthy aortic media (control), intima and media of FS, intima and media of FL) using the RNeasy® Tissue Mini Kit (Qiagen, les Ulis, France) according to the manufacturer's instructions. Reverse transcription was performed using the Maxima First Strand cDNA Synthesis kit (Thermo Scientific, Illkrich, France). Human PN-1, LRP-1, and t-PA mRNA levels were analyzed by real-time qPCR with SYBR Green detection (BioRad, Marnes-la-Coquette, France).

Primer sequences used to perform real-time qPCR are indicated in Table 2. The mRNA levels were normalized to human HPRT-1 (hypoxanthine phosphoribosyltransferase -1) mRNA.

**Table 2:** **Primer sequences used for qPCR analysis, HPRT-1: reference gene**.

**Gene**	**Forward**	**Reverse**
PN-1	5′-CCGCTGAAAGTTCTTGGCA-3′	5′-CAGCACCTGTAGGATTATGTCG-3′
t-PA	5′-TGCCTGCTCTGAGGGAA-3′	5′-TGCCTATCAGGATCATGGAAT-3′
LRP-1	5′-ATCCAACAGATCAACGACGA-3′	5′-TCCCAGCCACGGTGATAG-3'
HPRT-1	5′-TGAGGATTTGGAAAGGGTGT-3′	5′-GAGCACAGAGGGCTACAA-3′

### Western blot

Frozen pulverized aortic intimal and medial tissues or cultured vSMCs were homogenized in lysis buffer (50 mM Tris, 150 mM NaCl, 5 mM EDTA, 1% Triton-X, 0.1% SDS) containing a cocktail of protease inhibitors, P8340 (Sigma Aldrich, St Louis, MO, USA). The lysates were clarified by centrifugation (15, 000 g, 20 min at 4°C) and protein concentration of each sample was determined by BCA protein assay (Thermo Scientific). Proteins of human tissue (15 μg) or cultured vSMCs (4 μg) were separated by 4–20% (PN1) and 10% (LRP-1, t-PA) SDS-polyacrylamide gels under non-reducing conditions.

Proteins were transferred to a nitrocellulose membrane (Hybond, Amersham Biosciences, Illkrich, France) and then blocked in 5% low fat milk-PBS-T or 4% fish gelatin-PBS-T for 1 h. Membranes were incubated overnight at 4°C with the following primary antibodies: rabbit anti-human PN-1 (1:600, in house), mouse anti-human LRP-1 (1:250, Sigma-Aldrich), and mouse anti-human t-PA (1:250, Abcam). The secondary antibody used was a mouse (Sigma-Aldrich) or a rabbit IgG conjugated with peroxidase (Jackson ImmunoResearch Labs, PA, USA) for 1 h at room temperature. Detection by chemiluminescence was performed using the ECL+ (enhanced chemiluminescence) kit from Amersham Biosciences.

### Plasminogen activation and plasmin assay

One hundred microliters of conditioned medium from tissue of healthy media, intima, and media FS and FL was added in 96-well plates. vSMCs were grown to confluence and incubated with or without native or aggregated LDL for 48 h, in the presence of different concentrations of human plasminogen (0, 125, 250, 500 nM) from Cryopep (Montpellier, France). The plasmin selective fluorescent substrate was added [MeOSuc-Ala-Phe-Lys-AMC (trifluoroacetate salt), 40 μM; Bachem, Weil am Rhein, Germany] to the cells and conditioned medium. Fluorescence emission kinetics were measured at 460 nm after 380 nm excitation with a Tecan monochromator infinite®M200 pro. Plasmin formation was calculated from the rates of fluorescence emission/second. Each experiment was performed in triplicate.

### Cell biology

Primary human vSMC cultures were grown in 48-well plates or 8-well chamber slides and incubated with PN-1 alone (125 nM, in house), plasmin alone (25 nM, American diagnostic inc, Connecticut, USA) or PN-1 and plasmin together which were pre-incubated for 1 h at 37°C to allow complex formation, without or with RAP (Receptor associated protein) (50 μg/ml, Molecular innovation, Michigan, USA) and added to vSMCs for 2 h at 37°C.

First, cells were washed with PBS and fixed in PBS containing 3.7% paraformaldehyde, then permeabilized with 0.1% Triton X-100 (Sigma-Aldrich) in PBS. Cells were blocked with 5% BSA in PBS and incubated with the following primary antibodies: rabbit anti-human PN-1 (1:600, in house), mouse anti-LRP-1 (1:250, Sigma-Aldrich), and mouse anti-plasmin/plasminogen (1:50, Thermo Scientific); this latter was used to detect both plasmin alone and plasmin-PN-1 complexes. Alexa Fluor®488 and 555-labeled (Life Technologies) secondary antibodies were used for visualization. Finally cells were washed and incubated with DAPI (4, 6-diamidino-2-phenylindole) to stain the nuclei. Staining was visualized using a Leica fluorescence microscope or a Zeiss LSM 780 confocal microscope and images were analyzed with the Zen Lite 2012 software (France).

### Statistical analysis

Values are expressed as means ± SEM, the number of samples (*n*) is indicated in the figure legends. Since, despite the small number of experiments, point dispersion was limited, cell biology experiments are represented by histograms and the difference between groups was tested using a paired *t*-test. Tissue data are represented by dot plots and the differences between two medians tested using the non-parametric Mann-Whitney test (Prism 5, GraphPad software). Differences between groups are considered statistically significant when *p* < 0.05.

## Results

### Tissue biology of plasmin(ogen), PN-1, and LRP-1 in early stages of human atherosclerosis

The atherosclerotic lesions were characterized macroscopically and classified after histological examination by staining with hematoxylin & eosin or Masson's trichrome. The different stages of atherosclerotic lesions were determined according to the classification of Stary et al. (1995). Here we investigated two stages of early atheroma, fatty streaks (FS, type II) and fibrolipidic lesions, also named fibro-atheroma (FL, type IV).

**Figure 1:**
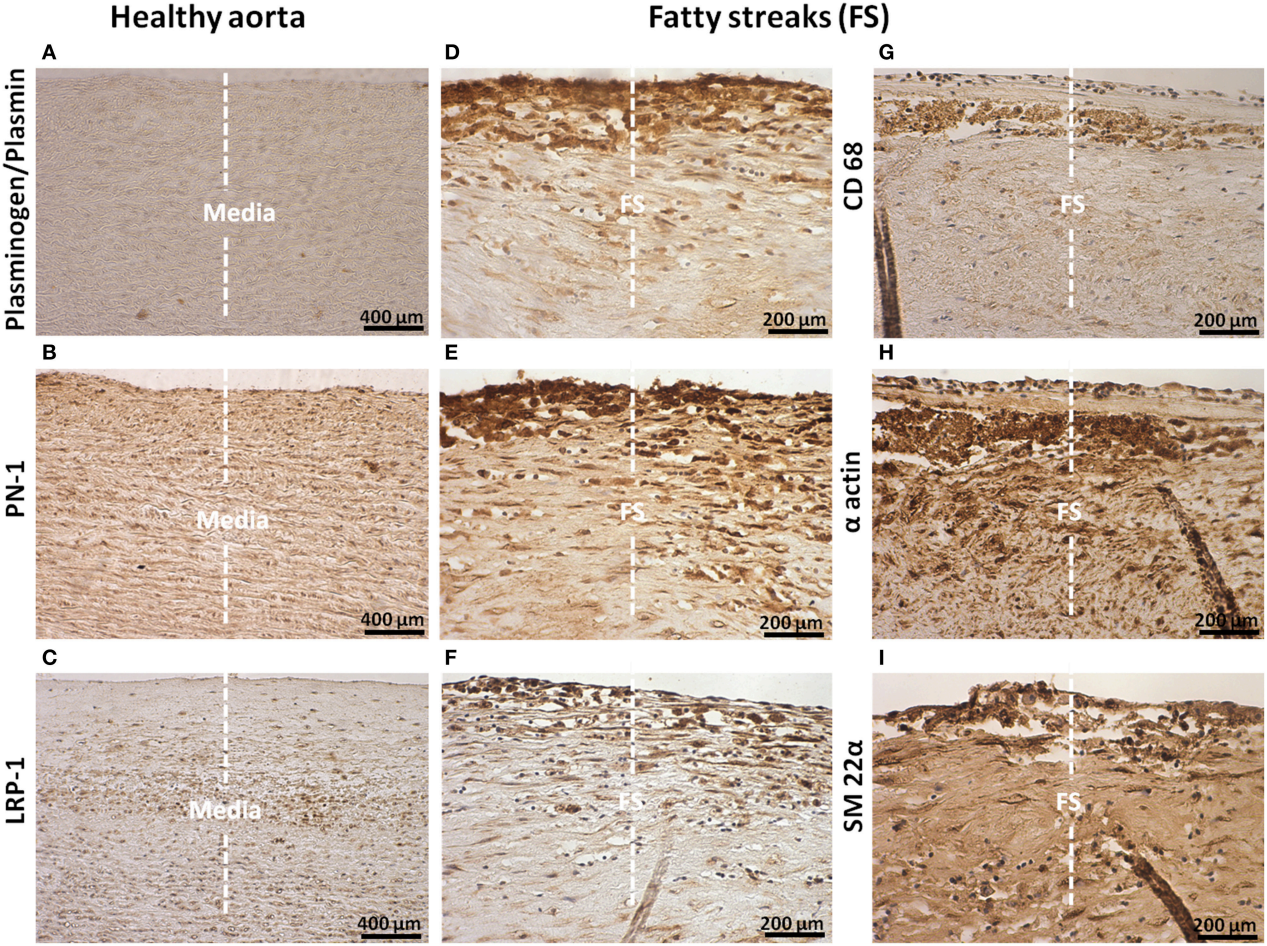
**Cellular localization of plasminogen/plasmin, PN-1, LRP-1, CD68, α actin, and transgelin (SM 22α) in intima of fatty streaks (FS)**. Histological sections of healthy aortas and serial sections of FS. Immunostaining of plasminogen/plasmin in healthy aortas **(A)** and FS **(D)**. Immunosignal of PN-1 in healthy aortas **(B)**, FS **(E)**. Immunosignal of LRP-1 in healthy aortas **(C)** and FS (**F)**. Phagocytosis marker (CD 68) staining **(G)**. Smooth muscle cell marker staining [(α actin, **H**) and (SM 22α, **I**)]. The negative control is shown in Figure 2A.

The presence of plasminogen/plasmin, PN-1, and LRP-1 in healthy aortas, and on serial sections of FS and FL, was analyzed by immunohistochemistry. In healthy aortas, no positive staining for plasminogen/plasmin was observed (Figure 1A). In contrast, staining of PN-1 and LRP-1 was weak and always associated with vSMCs in the media (Figures 1B,C).

Plasminogen/plasmin staining was detected in FS especially in intimal foam cells (phagocytes, Figure 1D). PN-1 immunostaining (Figure 1E) was intense and also associated with intimal foam cells as revealed by CD68 staining (Figure 1G) including vSMCs as revealed by vSMC markers, α actin (Figure 1H) and SM-22α (Figure 1I). As observed for PN-1, staining of LRP-1 was also increased in FS (Figure 1F) and associated with intimal foam cells.

**Figure 2:**
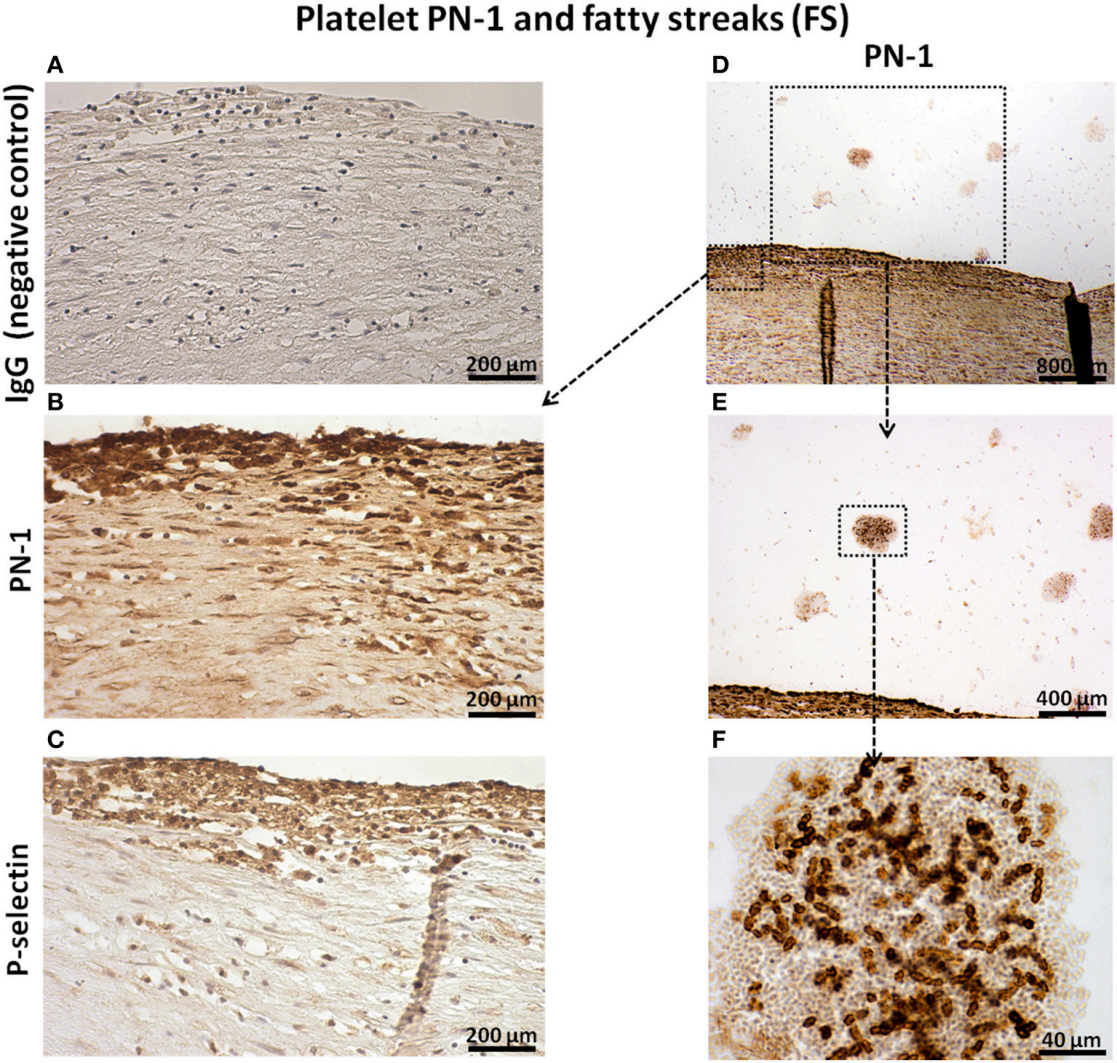
**Platelet PN-1 and fatty streaks (FS)**. Serial sections of FS. IgG (negative control, **A**). Immunosignal of PN-1 in FS **(B,D,E)** and in luminal platelet aggregates **(D,E,F)**. Immunosignal of p-selectin **(C)**.

PN-1 was also associated with intimal platelets and cells (Figures 2B,D) identified by P-selectin (Figure 2C). PN-1 was cell-associated but also cell-independent and potentially released by intraluminal fibrin-platelet aggregates as they contain large amounts of PN-1 (Figures 2D–F).

**Figure 3:**
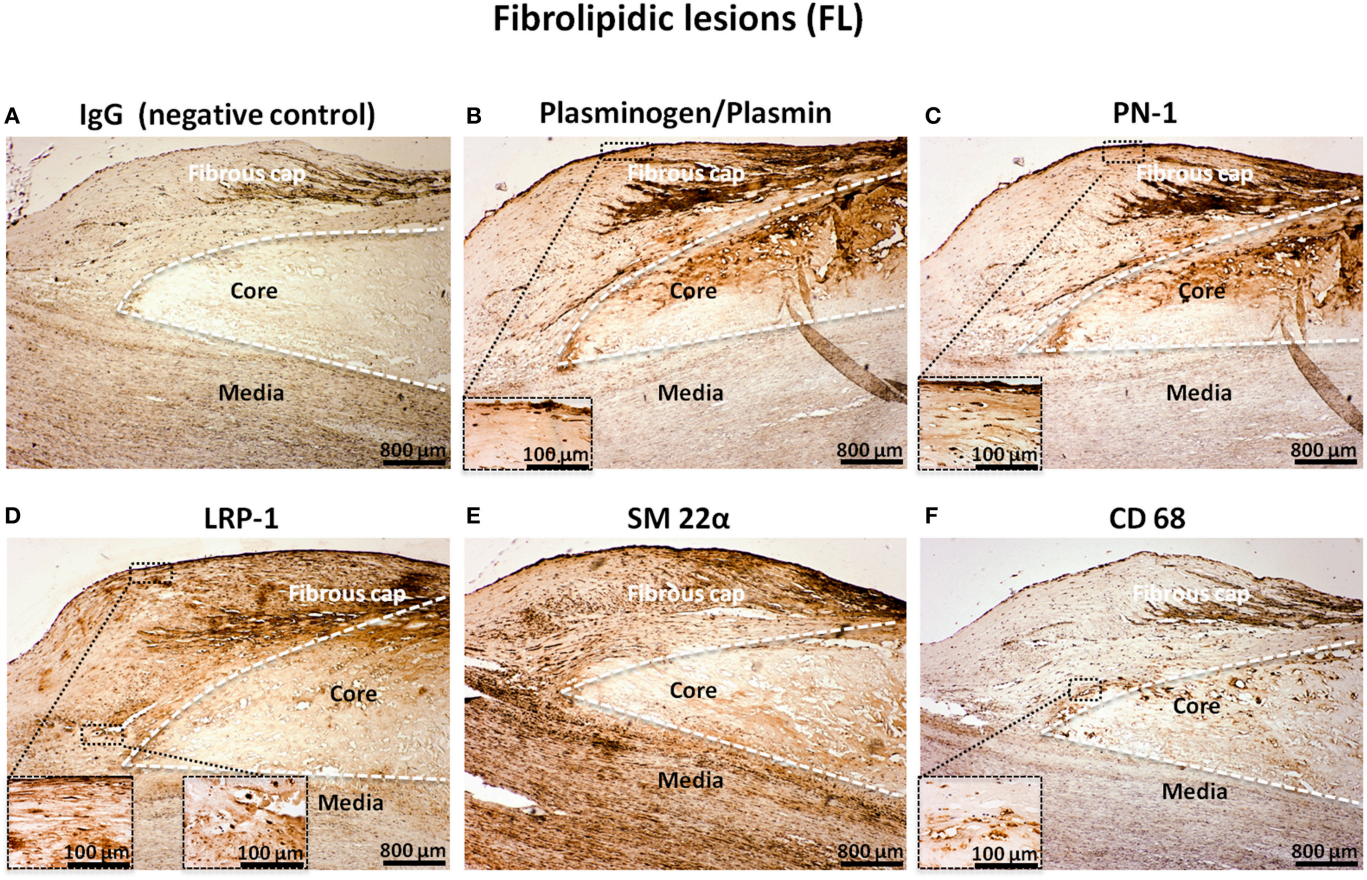
**Cellular localization of plasminogen/plasmin, PN-1, LRP-1, SM 22α, and CD68 in fibrolipidic lesions (FL)**. Serial sections of FL. IgG (negative control, **A**). Plasminogen/plasmin staining **(B)**. Immunosignal of PN-1 **(C)**. Immunostaining of LRP-1 **(D)**. Smooth muscle cell marker staining (SM 22α, **E**). Phagocytosis marker (CD 68) staining **(F)**.

In FL, the immunostaining of plasminogen (Figure 3B), PN-1 (Figure 3C), and LRP-1 (Figure 3D) was intense and associated with intimal cells including vSMCs in the fibrous cap as revealed by the vSMC marker, SM 22α (Figure 3E). The negative control is shown in Figure 3A. The acellular lipid core was positive for plasminogen/plasmin (Figure 3B) and was also occasionally positive for PN-1 (Figure 3C). LRP-1 was less observed in the acellular lipid core (Figure 3D). Phagocytes, revealed by CD68 were also observed in the lipid core (Figure 3F). LRP-1 (Figure 3D) could be associated with CD68 positivity (Figure 3F) particularly at the plaque shoulder.

**Figure 4:**
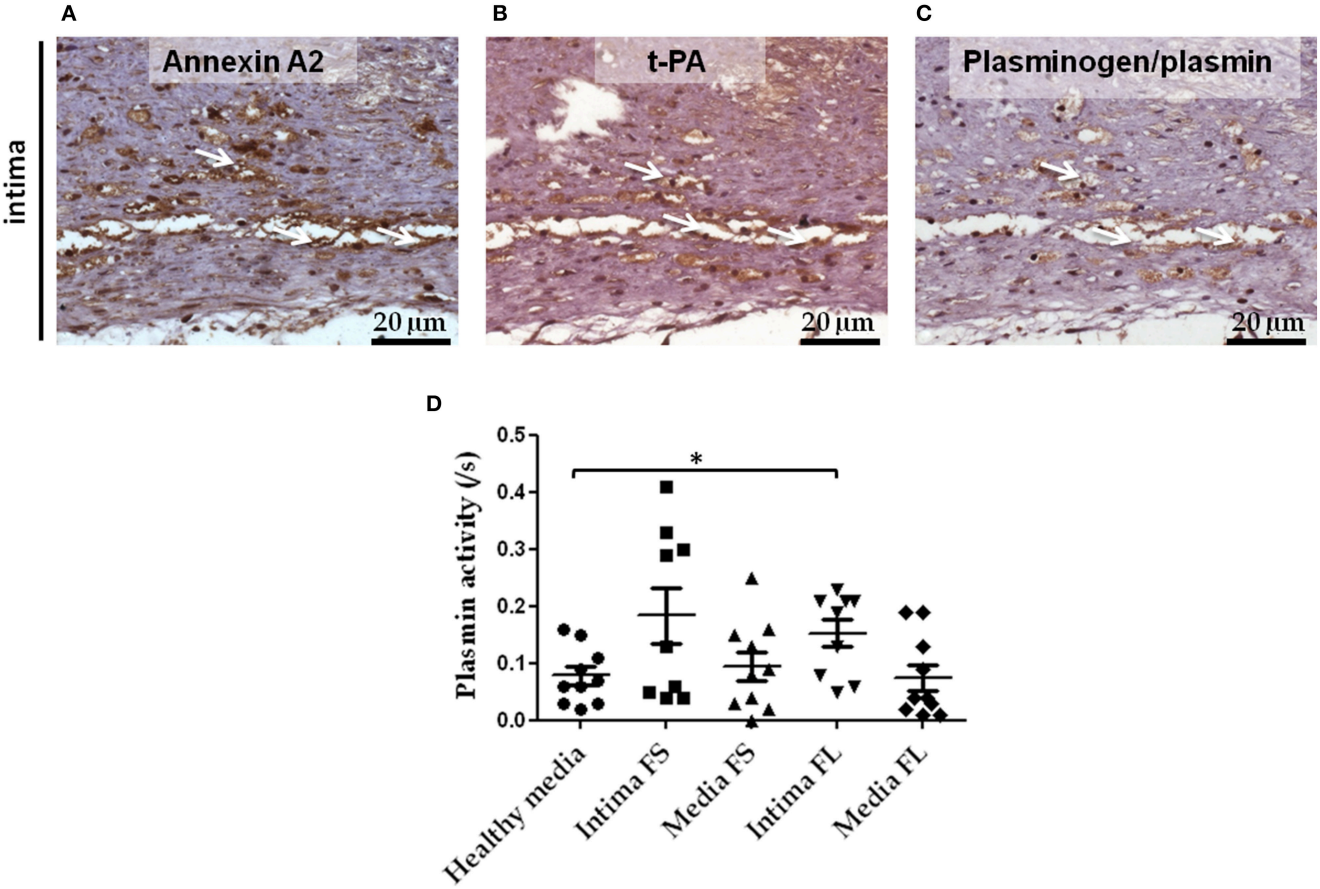
**Colocalization of Annexin A2, t-PA, and plasminogen in serial sections of FS intima**. Immunostaining of Annexin A2 **(A)**, t-PA **(B)**, and plasminogen **(C)** in FS. White arrows indicate the area in which Annexin A2, t-PA, and plasminogen immunostaining were colocalized. Plasmin activity in conditioned medium of healthy media, intima and media SL and FL **(D)**: healthy media *n* = 10 vs. intima of FL *n* = 9; ^*^p < 0.05. The non-parametric Mann-Whitney test was used.

Serial sections of FS were used to demonstrate the presence of proteins involved in the activation of the plasminergic system in early atheroma. We could indeed detect positive immunostaining for Annexin A2 (Figure 4A), t-PA (Figure 4B), and plasminogen (Figure 4C) in FS associated with intimal foam cells. These different proteins colocalized on the same platforms and therefore can catalyze the formation of active plasmin on the surface of cells. We also demonstrated an increase in plasmin activity in conditioned medium of intimal FL as compared to healthy media (Figure 4D).

**Figure 5:**
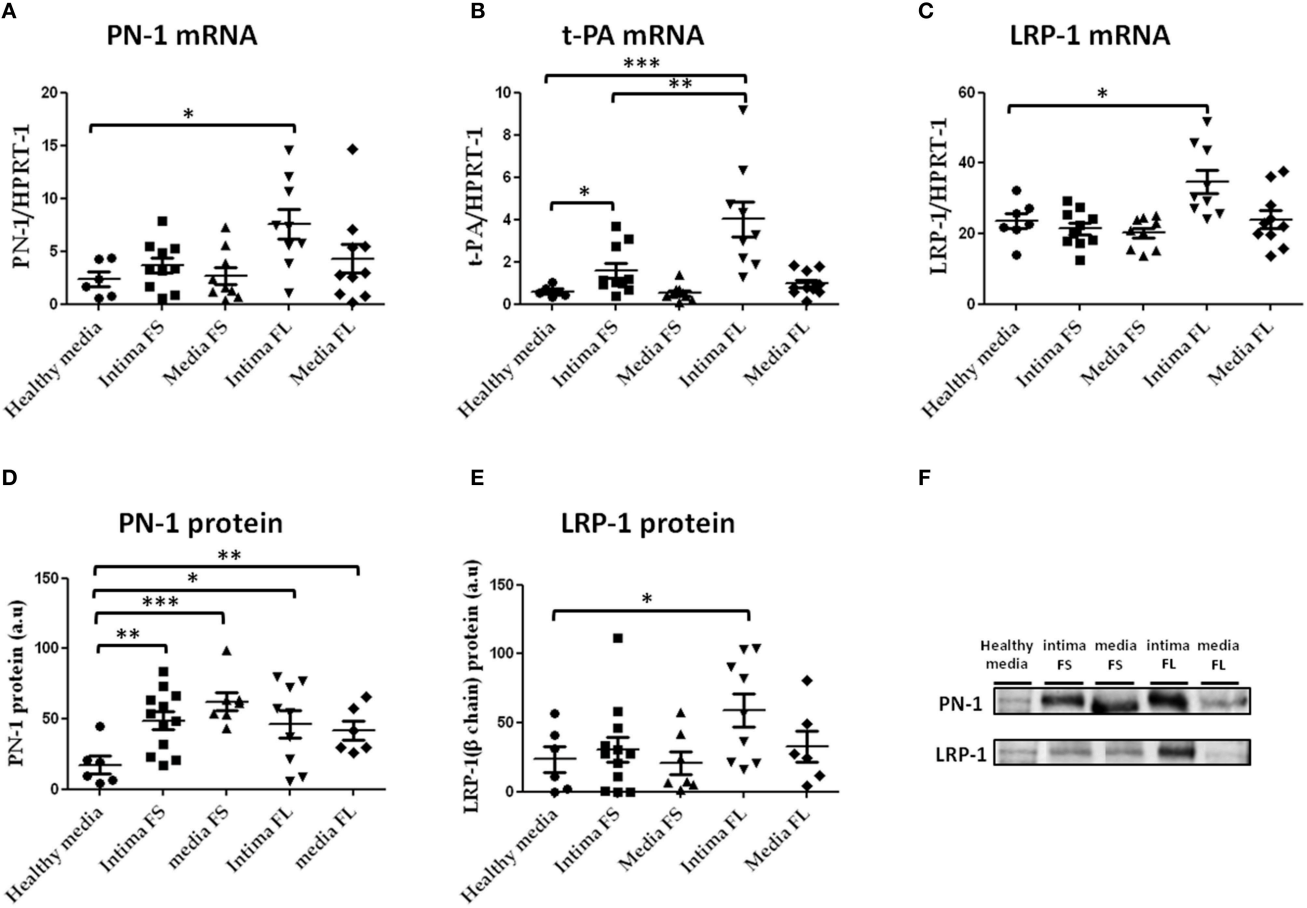
**PN-1 (A), tissue plasminogen activator (t-PA; B), and LRP-1(C) mRNA expression and PN-1 (D, F) and LRP-1 (E, F) protein expression in human aortic tissues (healthy aortas, intima and media of FS, intima and media of FL)**. PN-1 **(A)**: healthy media *n* = 6 vs. intima of FL *n* = 9; ^*^p < 0.05, t-PA **(B)**: healthy media *n* = 6 vs. intima of FL *n* = 9; ^***^p < 0.001, intima of FS *n* = 10 vs. intima of FL *n* = 9; ^**^p < 0.01, healthy media: *n* = 6 vs. intima of FS *n* = 10; ^*^p < 0.05. LRP-1 **(C)**: healthy media *n* = 7 vs. intima of FL *n* = 9; ^*^p < 0.05. PN-1 protein expression **(D,F)**: healthy media *n* = 6 vs. intima of FS *n* = 12; ^**^p < 0.01, vs. media of FS *n* = 7; ^***^p < 0.001, vs. intima of FL *n* = 9; ^*^p < 0.05, vs. media of FL *n* = 6; ^**^p < 0.01. LRP-1 protein **(E,F)**: healthy media *n* = 6 vs. intima of FL *n* = 9; ^*^p < 0.05. The non-parametric Mann-Whitney test was used. There was no loading control because intima of advanced lesions is acellular and there are no appropriate stable housekeeping proteins.

PN-1 (Figure 5A) and LRP-1 mRNA (Figure 5C) were increased in the intima of FL compared to healthy aortas, as was t-PA mRNA in the intima of both FS and FL compared to healthy aortas (Figure 5B). In contrast, plasminogen mRNA was not detectable in these tissues, demonstrating the plasma-borne origin of plasminogen detected by immunohistochemistry in FS and FL.

Western blot analysis of tissue extracts derived from healthy aortas and atherosclerotic plaques revealed that PN-1 protein (Figures 5D,F) was barely detectable in normal aortas and significantly accumulated in the intima and media of FS and FL.

The small difference between mRNA and protein extract suggests that part of the PN-1 was not synthesized *in situ*, but could be retained intracellularly.

Likewise, increased LRP-1 (Figures 5E,F) protein levels were observed in the intima of FL as compared to healthy aortas. In contrast, t-PA protein was undetectable in these aortic tissue extracts.

### vSMC biology

#### A- effect of native and aggregated (ag) LDL on plasmin activity and the expression of PN-1, t-PA, and LRP-1 in vSMCs

LRP-1 is a scavenger receptor involved in both clearance of serine protease-PN-1 complexes (Muhl et al., 2007) and in the internalization of LDL (Llorente-Cortes et al., 2007). Because of the modulation of the expression of PN-1, t-PA, and LRP-1 observed during the early stages of atheroma, we checked the effects of LDL and agLDL on the expression of PN-1, t-PA, and LRP-1 and the potential consequences on the activation of the fibrinolytic system.

For this purpose, we mimicked the pathological vascular condition by using primary vSMCs, pretreated or not with LDL or agLDL for 48 h.

**Figure 6:**
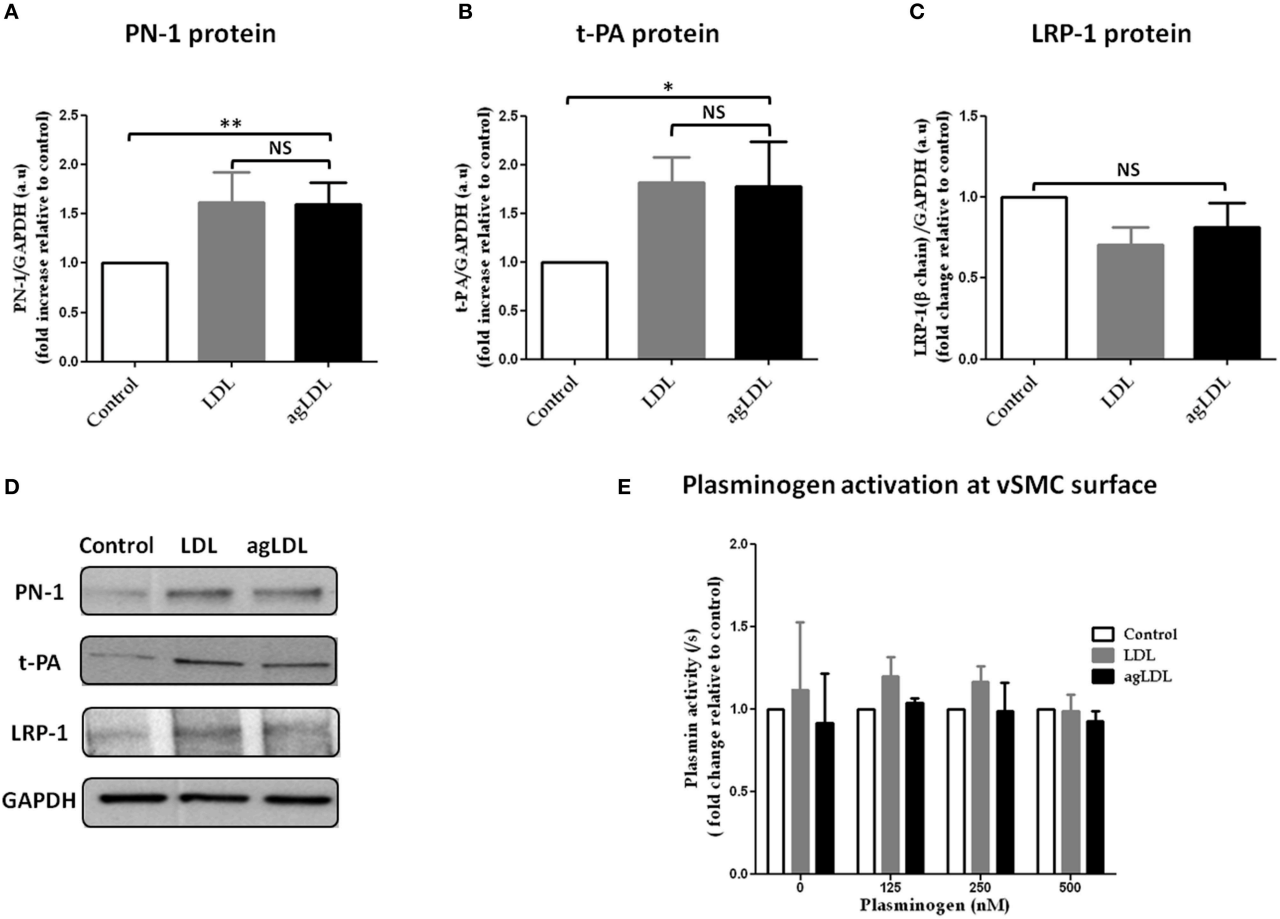
**Effects of native and aggregated (ag) LDL on PN-1 (A,D), t-PA (B,D), LRP-1 (C,D) protein expression and plasminogen activation (E) in vSMCs**. The results are expressed relative to control for each experiment. PN-1 protein expression **(A,D):** LDL 1.62 ± 0.30, agLDL 1.60 ± 0.21; ^**^p < 0.01, *n* = 5. t-PA protein expression **(B,D)**: LDL 1.82 ± 0.26, agLDL 1.78 ± 0.46; ^*^p < 0.05, *n* = 5. LRP-1 **(C,D)** protein expression (*n* = 5). The plasminogen activation (*n* = 3 and each experiment done in triplicate) at the surface of vSMCs **(E)**. The paired *t*-test was used.

Treatment of vSMCs with either LDL or agLDL induced overexpression of PN-1 protein as compared to control (Figures 6A,D). t-PA protein levels were also significantly increased in the presence of both LDL and agLDL (Figures 6B,D). In contrast, neither LDL nor agLDL affected LRP-1 protein expression in vSMCs (Figures 6C,D).

The effect of LDL and agLDL on plasminogen activation was investigated at the surface of vSMCs. Neither LDL nor agLDL had any relevant effect on plasminogen activation whatever the concentration of exogenous plasminogen added to the vSMCs (Figure 6E).

#### B- plasmin-PN-1 complexes are internalized by LRP-1 in vSMCs, whereas plasmin alone is not internalized

**Figure 7:**
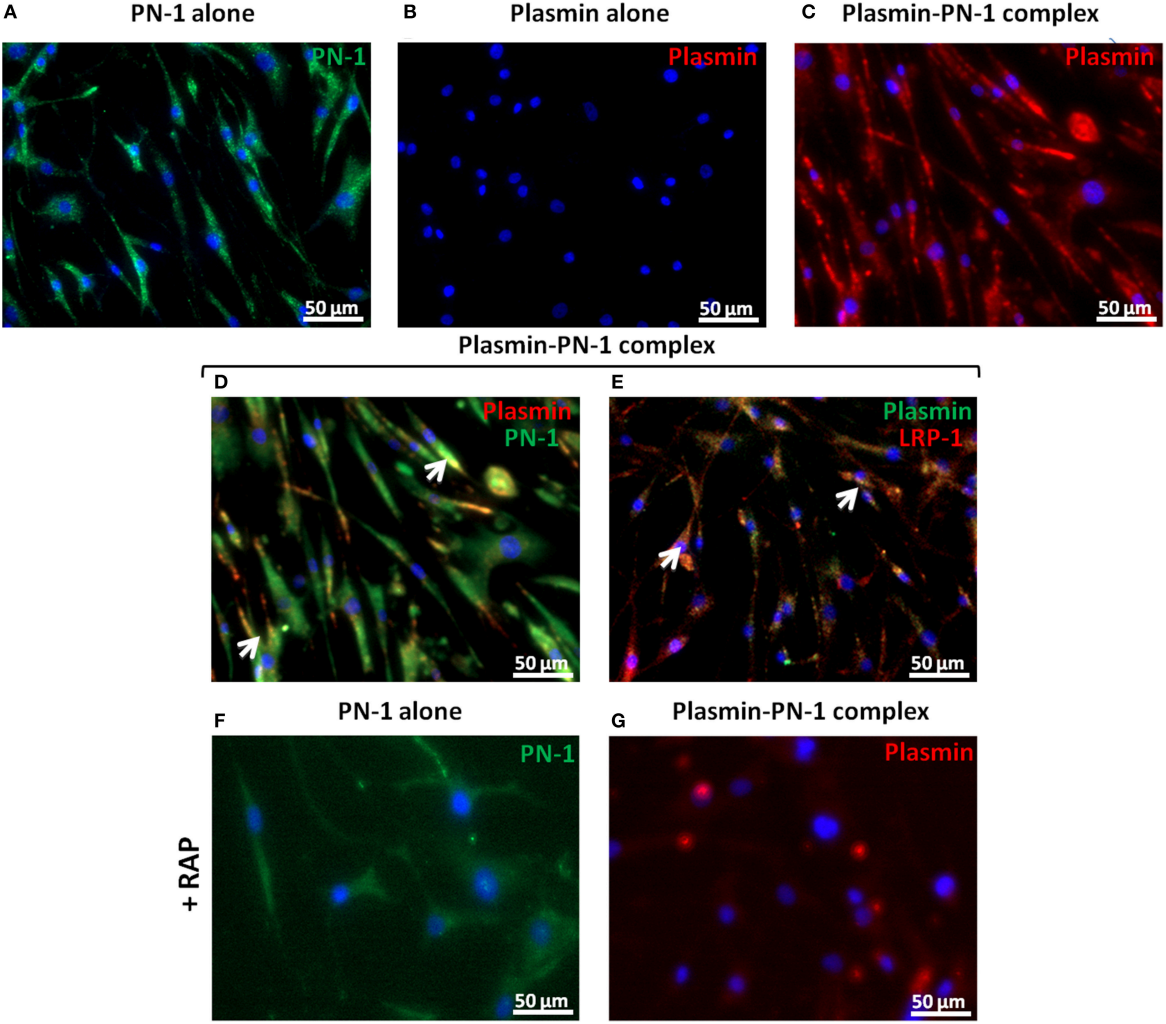
**Internalization of PN-1, plasmin, and plasmin-PN-1 complexes in vSMCs**. PN-1 alone (125 nM, **A,F**), plasmin alone (25 nM, **B**), and pre-formed plasmin-PN-1 complexes (**C**,**D**,**E,G**) were added to vSMCs for 2 h at 37°C without (**A–E**) or with RAP (50 μg/ml**, F,G**). After cell permeabilization, PN-1 alone (**A,F**) was detected by Alexa Fluor®488-labeled secondary antibody (green), plasmin alone (**B**) by Alexa Fluor®555-labeled secondary antibody (red) and nuclei by DAPI staining (blue). Plasmin-PN-1 complexes were revealed with plasmin antibody and detected by Alexa Fluor®555-labeled secondary antibody (**C,G**, red) or PN-1 and plasmin antibodies and detected with Alexa Fluor®488 (**D**, green) and 555-labeled secondary antibody (**D**, red) respectively. Yellow color and white arrows highlight examples of intracellular co-localization of plasmin-PN-1 complexes revealed with PN-1 and plasmin antibodies (**D**). LRP-1 was detected with Alexa Fluor®555-labeled secondary antibody (**E**, red), plasmin-PN-1 complexes were revealed with plasmin antibody and detected by Alexa fluor®488-labeled secondary antibody (**E**, green). Yellow color and white arrows indicate the intracellular colocalization of LRP-1 and plasmin-PN-1 complexes (**E**).

**Figure 8:**
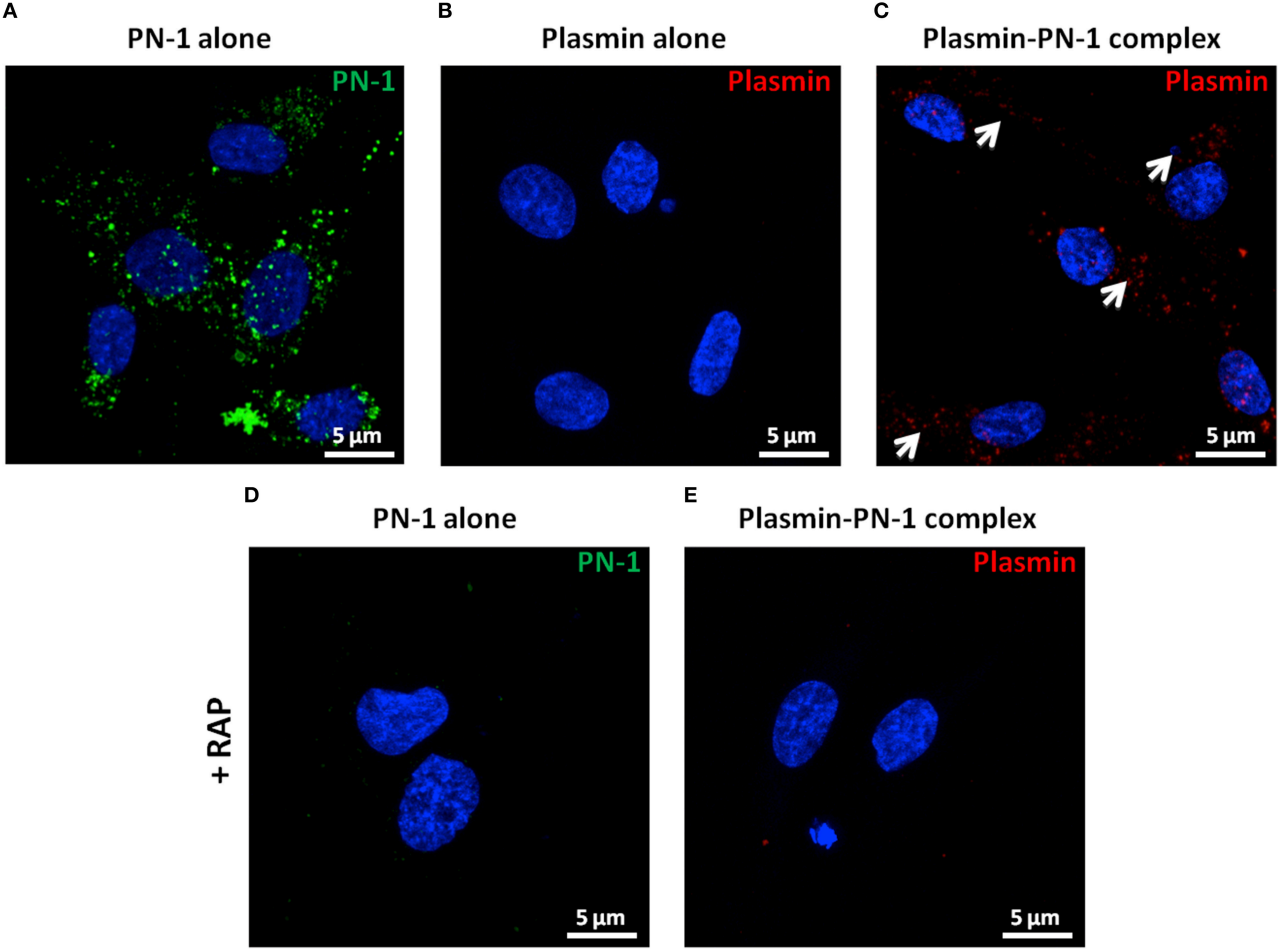
**Internalization of PN-1, plasmin, and plasmin-PN-1 complexes in vSMCs (confocal microscopy)**. PN-1 alone (125 nM, **A,D**), plasmin alone (25 nM, **B**), and pre-formed plasmin-PN-1 complexes (**C**,**E**) were added to vSMCs for 2 h at 37°C without (**A–C**) or with RAP (50 μg/ml, **D,E**). After cell permeabilization, PN-1 alone (**A, D**) was detected by Alexa Fluor®488-labeled secondary antibody (green), plasmin alone (**B**) and plasmin-PN-1 complexes (**C,E**) by Alexa Fluor®555-labeled secondary antibody (red) and nuclei by DAPI staining (blue). White arrows indicate the intracellular localization of plasmin-PN-1 complexes (**C**). The images are presented as maximum intensity projection of confocal microscopy.

PN-1 is known to be able to neutralize the enzymatic activities of serine proteases by forming covalent complexes that can be cleared by LRP-1 in the arterial wall. Therefore, we analyzed the internalization of plasmin and PN-1 by LRP-1 in human primary vSMC cultures by immunofluorescence and confocal microscopy. The addition of PN-1 alone to vSMCs leads to an intracellular accumulation of PN-1 (Figures 7A, 8A; green), whereas the addition of plasmin alone was not followed by its internalization by vSMCs (Figures 7B, 8B; red).

Plasmin-PN-1 complexes detected with an anti-plasmin antibody (Figures 7C, 8C; red) or with both anti-PN-1 (green) and anti-plasmin (red) antibodies (Figure 7D) were internalized and accumulated in the cytosolic compartment. Co-staining of plasmin-PN-1 complexes (Figure 7E; green) and LRP-1 (Figure 7E; red) shows the colocalization and the intracellular accumulation of the protease, the serpin and the scavenger receptor in vSMCs.

Incubation with RAP (Figures 7F,G, 8D,E), known as a LRP-1 antagonist, inhibited the internalization of PN-1 or plasmin-PN-1 complexes in vSMCs.

## Discussion

Previous studies demonstrated that plasma proteins, such as LDL, are radially convected through the arterial wall and retained by its components (Tabas et al., 2007), and that the fibrinolytic system is activated *in vitro* in contact with vSMCs (Meilhac et al., 2003) and *in vivo* during development of atherosclerotic plaques (Bauriedel et al., 1999; Rossignol et al., 2006). In this study, we explored the source, the activation, and the uptake of plasmin in the arterial wall of early atheroma. Since plasminogen is not synthesized within the arterial wall, circulating plasminogen is delivered to it by transmural convection from plasma according to the pressure gradient existing between the lumen and the adventitial layer (Tedgui and Lever, 1987; Michel et al., 2007). We showed that plasminogen, t-PA, and Annexin A2 colocalize in the same area of early atheroma, providing evidence of the existence of a vSMC platform able to activate plasminogen within the wall. In contrast to healthy aortas, we showed that an active fibrinolytic system is present in early atheroma. Plasminogen and t-PA potentially form quaternary complexes with the Annexin A2-S100 protein tetramer on the vSMC surface, which acts as a bridge between plasminogen and t-PA leading to the production of active plasmin (Madureira et al., 2011). We also showed the presence of proteolytic stress caused by plasmin in the intima of early atheroma. The proteolytic activity of cell-mediated plasmin generation could participate in atherosclerotic development, vSMC migration and rarefaction in the arterial wall (Torzewski et al., 2004; Rossignol et al., 2006).

PAI-1 and PN-1 are the principal tissue serpins able to block the plasminergic system in the arterial wall at different levels (Bouton et al., 2012). Contrasting with PAI-1 and α2-antiplasmin, PN-1 is not diffusible, and is the main serpin within the arterial wall, always associated with GAGs and barely detectable in the plasma (Richard et al., 2006; Bouton et al., 2012). PN-1 is a key cell-associated serpin expressed by platelets, macrophages (Mansilla et al., 2008), endothelial cells, vSMCs, and fibroblasts (Bouton et al., 2012). In this study, we investigated the source of PN-1 in early human atheroma and the ability of vSMCs to internalize plasmin-PN-1 complexes. Using human FS and FL, we identified both adherent platelets and intimal foams cells, including vSMCs, as the main sites of PN-1 presence in early atheroma.

Our results also showed an increase in both mRNA and protein levels of PN-1 associated with the intima in early human atheroma. Plasmin is one of the main proteases responsible for the release of active TGF-β (Jenkins, 2008) which is a powerful inducer of PN-1 expression (Gomez et al., 2013). Therefore, TGF-β could be a link between plasmin and PN-1 in early atheroma. In this context, PN-1 is not only overexpressed by intimal foam cells, but also released by aggregated platelets and therefore retained by the GAG-rich intima (Wight, 1985). These results are in agreement with those showing that PN-1 expression correlates with the different stages of evolution of atherosclerosis (Mansilla et al., 2008) and the fact that subendothelial adherent platelets are early initial events in atheroma (Massberg et al., 2002). Mansilla *et al* (Mansilla et al., 2008) suggested that the major sources of PN-1 in vulnerable plaques were platelets and observed a considerable enrichment of PN-1 in phagocytes (CD68^+^ cells). vSMCs can also acquire a macrophage-like phenotype due to their plasticity, including CD68 positivity associated with phagolysosome activities (Lacolley et al., 2012; Michel et al., 2012). Therefore, foams cells, including vSMCs, can potentially assume the role of endocytosis of plasmin-PN-1 complexes in the arterial wall.

LRP-1 is a member of the LDL scavenger receptor family implicated in the uptake of multiple ligands (Boucher and Gotthardt, 2004).

LRP-1 is expressed in normal healthy vascular tissues, associated with vSMCs in the media of mammary arteries, suggesting that it is involved in physiological processes (Lupu et al., 1994). LRP-1 is also highly expressed within human atherosclerotic lesions (Luoma et al., 1994).

Our immunohistochemical and biochemical data obtained in FS and FL show that the expression of the scavenger receptor, LRP-1, is increased in these lesions, and is associated with intimal foam cells. Previous studies showed that LRP-1 was up-regulated in macrophage-derived foam cells and proliferating vSMCs in the aorta of rabbits fed a high-cholesterol diet, two cell types involved in the development of atherosclerotic lesions (Watanabe et al., 1994; Boucher and Gotthardt, 2004). The use of mice deficient in SMC LRP (LRP^smc−∕−^) associated with LDLR knockout (LDLR^−∕−^) resulted in a severe vSMC proliferation within the aorta, disruption of elastic laminae, and enhancement of atherosclerosis, suggesting that vSMC LRP plays an important atheroprotective role via clearance capacities not limited to LDL endocytosis alone (Boucher et al., 2003).

vSMCs are the stromal cells of the arterial wall. They support the initial lipid injury by the uptake and retention via glycosaminoglycans of LDL, leading to foam cell formation in the intimal layer of early atheroma (Parker and Odland, 1966; Goldstein et al., 1977; Katsuda et al., 1992; Kockx et al., 1998; Owens et al., 2004; Michel et al., 2012; Allahverdian et al., 2014; Feil et al., 2014; Shankman et al., 2015). LRP-1 is implicated in the selective uptake of cholesteryl esters of aggregated LDL and the formation of lipid storage droplets in human arterial vSMCs (Llorente-Cortes et al., 2006, 2007) and monocyte-derived macrophages (Llorente-Cortes et al., 2007). We observed that PN-1 and LRP-1 are up-regulated in the intima of early atheroma. The LDL uptake by vSMCs (Llorente-Cortes et al., 2006) could induce, at least in part, the overexpression of PN-1 and LRP-1 that we observed in early atheroma. Our data show that treatment of cultured vSMCs from healthy aortas with LDL for 48 h increased protein levels of PN-1 and t-PA, whereas it has no effect on LRP-1 protein, suggesting that LRP-1 expression is constitutive in vSMCs whereas PN-1 and t-PA are inducible. Moreover, we observed no change in plasmin generation after incubation of cultured vSMCs with LDL and exogenous plasminogen.

Besides its function in the selective uptake of LDL (Llorente-Cortes et al., 2007), LRP-1 is also implicated in endocytosis of protease-PN-1 complexes, and GAGs enhance the internalization of thrombin-PN-1 complexes by stromal cells, leading to their intracellular lysosomal degradation (Knauer et al., 1997, 1999; Crisp et al., 2000; Lillis et al., 2008).

We showed that PN-1 alone and plasmin-PN-1 complexes were internalized by cultured vSMCs, whereas plasmin alone was not. Co-staining of plasmin-PN-1 complexes and LRP-1 shows the intracellular colocalization of the receptor and its ligands in the vSMCs. The use of the specific LRP inhibitor, RAP, indicated that plasmin-PN-1, and PN-1 alone are internalized via LRP-1 in vSMCs.

This result is in accordance with Muhl et al. (2007) who showed that application of FSAP (Factor VII activating protease)-PN-1 complexes to vSMCs of mice resulted in the intracellular accumulation of the complexes, but that FSAP alone was not internalized and that the uptake of this serine protease was a PN-1- and LRP-1-dependent mechanism.

## Conclusion

Our study showed that the plasminergic system is activated within the arterial intima in early atheroma. vSMCs and platelets are the main sources of PN-1 in human atherosclerotic lesions, but the endocytosed form of PN-1 is observed, at least partly, in intimal foam cells. These data suggest that tissue serpins could participate in the clearance capacities of the arterial wall. We also showed that intimal LDL uptake, which is a key process in the pathophysiology of early atheroma, could be responsible for the up-regulation of PN-1 levels in vSMCs. We demonstrated that human aortic vSMCs internalize plasmin-PN-1 complexes and neutralize the deleterious effects of plasmin on vSMCs (Meilhac et al., 2003; Rossignol et al., 2004) via the scavenger receptor LRP-1.

These results suggest that tissue PN-1 could limit, at least in part, the plasmin-dependent proteolytic injury of the wall by directly inhibiting plasmin, and by uptake of plasmin-PN-1 complexes. Despite this potential protective effect of PN-1, plasmin activity remains abnormally high in the intima of early atheroma.

Finally, being the main structural and functional stromal cell component of the arterial wall, vSMCs are also involved in the activation, inhibition, and internalization of blood-borne proteases by the arterial wall.

## Author contributions

Design of study: KB, JM, and MB; conducting the experiments: KB, LL; data analysis: KB; interpretation of the study data: KB, RB, LB, YB, BH, VA, MB, and JM; writing and critical revision of the manuscript: KB, RB, LB, YB, BH, VA, MB, and JM; final approval of the version to be published: KB, RB, LB, LL, YB, BH, VA, MB, and JM. KB, RB, LB, LL, YB, BH, VA, MB, and JM all agree to be accountable for all aspects of the work in ensuring that questions related to the accuracy or integrity of any part of the work are appropriately investigated and resolved.

## Funding

This work was supported by INSERM, Paris Diderot University and a grant from the French National Research Agency [grant number ANR-12-BSV1-0009 NEX-STARWall].

### Conflict of interest statement

The authors declare that the research was conducted in the absence of any commercial or financial relationships that could be construed as a potential conflict of interest.

## Abbreviations

Ag: aggregated
DAPI, 4: 6-DiAmidino-2-PhenylIndole
FL: Fibrolipidic Lesions
FS: Fatty Streaks
GAGs: GlycosAminoGlycans
LDL: Low Density Lipoprotein
LDLR: LDL receptor
LRP-1: LDL receptor-related protein-1
PAI-1: Plasminogen Activator Inhibitor-1
PN-1: Protease Nexin-1
RAP: Receptor Associated Protein
TGFβ: Transforming Growth Factor β
TIMPs: Tissue Inhibitors of MetalloProteinases
t-PA: tissue Plasminogen Activator
vSMCs: vascular Smooth Muscle Cells.
